# Ultrasound-based withdrawal of biologics in rheumatoid arthritis (RA-BioStop)

**DOI:** 10.1136/rmdopen-2026-007003

**Published:** 2026-08-07

**Authors:** Christian Dejaco, Irina Gessl, Rusmir Husic, Gregor Mitter, Thomas Deimel, Angelika Lackner, Gabriela Supp, Josef Hermann, Josef Smolen, Martin H Stradner, Daniel Aletaha, Gerold Schwantzer, Peter Mandl

**Affiliations:** 1 Department of Rheumatology and Immunology, Medical University of Graz, Graz, Austria; 2 Department of Rheumatology, Hospital of Brunico (SABES-ASDAA), Brunico, Italy; 3 Division of Rheumatology, Medical University of Vienna, Vienna, Austria; 4 Institute for Medical Informatics, Statistics and Documentation, Medical University of Graz, Graz, Austria; 5 Ludwig Boltzmann Institute for Arthritis and Rehabilitation, Vienna, Austria

**Keywords:** Rheumatoid Arthritis, DMARD, Ultrasonography, Clinical Trial, Biological Therapy

## Abstract

**Objective:**

To test whether ultrasonography of patients with rheumatoid arthritis (RA) in the American College of Rheumatology/the European Alliance of Associations for Rheumatology (ACR/EULAR) remission is useful for prediction of relapse after discontinuation of a biological disease-modifying antirheumatic drug (bDMARD).

**Methods:**

Prospective trial including patients with RA in persistent ACR/EULAR remission treated with conventional synthetic DMARDs plus bDMARDs. After stopping the bDMARD, nine study visits including clinical examination and ultrasound of 14 joints (blinded to clinical data) were conducted within 52 weeks. The primary hypothesis was that a Power Doppler (PD) score≥1 predicted relapse occurring within week 16 after bDMARD cessation. Relapse was defined as a change from remission to moderate/high disease activity according to the Simplified Disease Activity Score.

**Results:**

Inclusion of 110 patients was originally planned; however, the study was terminated after reaching 38 (34.5%) patients due to insufficient recruitment. Relapses till week 16 were numerically more common in patients with PD score≥1 at baseline than in those with PD=0 (9/30 (30.0%) vs 0/7 (0%), p=0.160). Similar observations were made for weeks 24 and 52. PD scores were higher at the time of relapse than at preceding visits (mean PD score difference: 3.2 (±4.5) points, p=0.034). There were trends towards a higher baseline PD score in patients who had a relapse until week 16 as compared with those who remained in remission (5.2±5.8 vs 2.3±3.0, p=0.079).

**Conclusion:**

The primary endpoint was not reached. The presented data should be interpreted as hypothesis-generating, serving to stimulate future research on the value of ultrasound in assessing remission and predicting relapse in RA.

**Trial registration number:**

NCT01602302.

**WHAT IS ALREADY KNOWN ON THIS TOPIC**

- Withdrawal of a disease-modifying antirheumatic drug is feasible in some patients with rheumatoid arthritis (RA) in clinical remission. However, no reliable predictors of flare have been identified yet.

**WHAT THIS STUDY ADDS**

- Patients with Power Doppler (PD) score≥1 showed a numerically higher relapse rate in comparison to PD-negative patients. Furthermore, patients yielded a higher PD score at relapse as compared with preceding visits in clinical remission.

**HOW THIS STUDY MIGHT AFFECT RESEARCH, PRACTICE OR POLICY**

- This study is hypothesis-generating, serving to stimulate further research on the potential value of ultrasonography as a predictor of relapse after cessation of a biological drug in patients with RA in clinical remission.

## Introduction

 Several studies have been conducted to identify the best treatment strategy to achieve remission in patients with rheumatoid arthritis (RA); however, much less is known about appropriate management once this status has been achieved.^1–5^ In patients with RA, ultrasound has been demonstrated to be superior to clinical examination for the detection of (residual) joint inflammation. Power Doppler (PD) signals were observed in up to 60% of patients with RA in clinical remission and conversely, sonographic signs of inflammation were absent in 20% of cases despite increased disease activity scores.^6–10^ Several studies have investigated whether sonographic findings may support decisions regarding biological disease-modifying antirheumatic drug (bDMARD) withdrawal, yielding conflicting results that are partly attributable to heterogeneous study design and the use of different remission criteria.^7 11–17^ Whether PD-positive synovitis may serve as a reliable predictor of future relapse in patients with RA fulfilling the American College of Rheumatology/the European Alliance of Associations for Rheumatology (ACR/EULAR) remission criteria who discontinue bDMARD therapy remains uncertain and was therefore investigated in the present study.

## Patients and methods

This was a prospective, phase IV trial involving two tertiary care centres in Austria (Medical University of Graz and Medical University of Vienna).^18 19^ We included consecutive patients with RA in persistent ACR/EULAR remission who were treated with a combination of csDMARDs plus bDMARDs at a stable dose. The full list of inclusion and exclusion criteria as well as allowed and prohibited medication are detailed in the study protocol (online supplemental file 1), as well as in online supplemental table 1. The study was registered at clinicaltrials.gov (NCT01602302).

### Study procedures

After inclusion, the bDMARD was stopped while treatment with csDMARD(s) was continued. Patients experiencing a relapse were classified as discontinuation failures and terminated the active phase of the study. These patients were subsequently treated according to routine clinical care^20^ but were still followed up until week 52.

At each visit, patients underwent clinical assessment including 28-joint tender joint (TJ) and swollen joint (SJ) counts, blood analysis and ultrasound examination of 14 joints. The selection of joints was based on the German U7 score, which in its original version is limited to the dominant side.^21–23^ Sonographic evaluations were performed immediately after clinical evaluation by independent investigators at each centre and included grey-scale synovitis (GSS) and PD assessment with semiquantitative scoring (0–3) according to Outcome Measures in Rheumatology (OMERACT) definitions.^24^ The investigator performing ultrasound was blinded to all clinical data and was not involved in any other part of the study. Additional details are shown in the study protocol (online supplemental file 1) and in online supplemental table 1.

### Endpoints

The primary endpoint was the proportion of patients with a relapse, defined as a change in disease status from ACR/EULAR remission to moderate or high disease activity (corresponding to a Simplified Disease Activity Score>11) between baseline and week 16 comparing patients with and without a PD score≥1 at baseline.^25 26^

Secondary endpoints included the proportion of patients with a relapse until week 24 and week 52, time to relapse, PD score at the visit preceding a clinical relapse as compared with the relapse visit, the predictive values of baseline PD and GSS score as well as the number of residual SJ or TJ at baseline for the occurrence of a relapse (until weeks 16, 24 or 52).

Other secondary endpoints, including blood biomarkers and radiographic progression, listed in the study protocol were not investigated due to early termination of the study.

**Figure 1:**
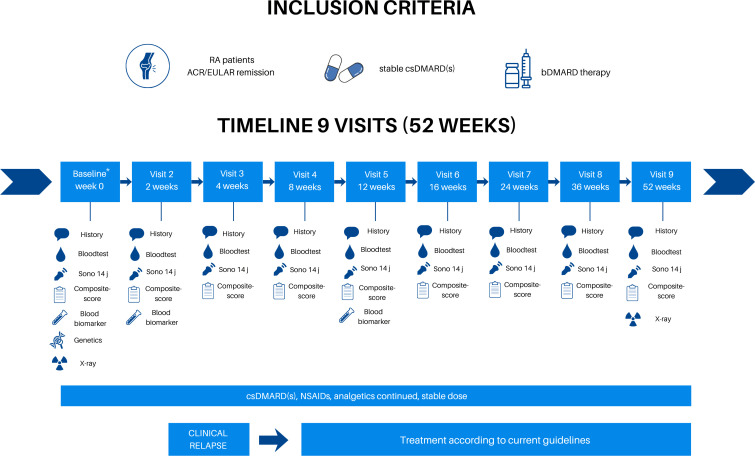
Key inclusion criteria, timeline and study procedures. ACR, American College of Rheumatology; bDMARD, biological disease modifying antirheumatic drug; csDMARD, conventional synthetic disease modifying antirheumatic drug; EULAR, European Alliance of Associations for Rheumatology; NSAID, non-steroidal antirheumatic drug; RA, rheumatoid arthritis; sono 14J, sonography of 14 joints.

See figure 1 and the study protocol (online supplemental file 1) for details concerning secondary endpoints, power calculation as well as statistical analyses.

## Results

### Clinical characteristics

40 patients with RA were recruited; 38 were included in the final analysis corresponding to 34.5% of the 110 patients foreseen by the protocol. The recruitment period lasted 7 years but ultimately, the study was terminated because of low participation. All nine study visits were attended by 23/38 (60.5%) patients. Patient characteristics and drug administration intervals are depicted in table 1 and online supplemental tables 3 and 4. No serious adverse events occurred, as shown in online supplemental file 2.

**Table 1:** Patient characteristics at baseline

Variable	Value	N
Female, n (%)	28 (73.7)	38
Age (years), mean (SD)	59.0 (12.1)	38
Disease duration (years), mean (SD)	12.8 (9.4)	38
Duration of remission (months), mean (SD)	20.3 (13.1)	38
Current smoker, n (%)	12 (31.6)	38
Previous smoker, n (%)	12 (31.6)	38
Packyears, median (range)	12.5 (0.0–70.0)	22
RF+* at diagnosis, n (%)	16 (69.6%)	23
RF+* at baseline, n (%)	15 (88.2%)	17
Anti-CCP+* at diagnosis, n (%)	20 (90.9)	22
Anti-CCP+* at baseline, n (%)	20 (95.2)	21
Tender joint count (28 joints), median (range)	0 (0–2)	38
Swollen joint count (28 joints), median (range)	0 (0–1)	38
Patient pain assessment (VAS) (mm), median (range)	2.5 (0–40)	38
Patient global assessment (VAS) (mm), median (range)	3.0 (0–40)	38
Evaluator global assessment (VAS) (mm), median (range)	0.0 (0–20)	38
CRP (mg/L), mean (SD)	2.6 (3.8)	38
ESR (mm/hour), mean (SD)	16.5 (13.8)	35
DAS-28, mean (SD)	1.9 (0.9)	35
CDAI, mean (SD)	1.3 (1.7)	38
SDAI, mean (SD)	1.6 (1.7)	38
HAQ, mean (SD)	0.17 (0.26)	28
Biologic DMARDs at time of inclusion into the study Infliximab, n (%) Etanercept, n (%) Adalimumab, n (%) Golimumab, n (%) Certolizumab pegol, n (%) Abatacept, n (%) Tocilizumab, n (%)	4 (10.5) 11 (28.9) 9 (23.7) 7 (18.4) 4 (10.5) 2 (5.3) 1 (2.6)	38
Conventional synthetic DMARDs at baseline Leflunomide, n (%) Methotrexate, n (%) Methotrexate+sulfasalazine, n (%)	8 (21.1) 27 (71.1) 1 (2.6)	38
No csDMARD, n (%)†	2 (5.3)	

* Cut-off for a positive value according to the local laboratory was 16 U/mL for RF and 10 U/mL for anti-CCP antibodies.

† Two patients did not receive csDMARD, which was noted as a protocol violation.

.CCP, anticyclic citrullinated peptide antibodies; CDAI, clinical disease activity index; CRP, C reactive protein; csDMARD, conventional synthetic disease modifying antirheumatic drug; DAS-28, Disease Activity Score 28; DMARD, disease modifying antirheumatic drug; ESR, erythrocyte sedimentation rate; HAQ, Health Assessment Questionnaire; RF, rheumatoid factor; SDAI, simplified disease activity index; VAS, Visual Analogue Scale.

### Ultrasound for relapse prediction

The overall mean (SD) GSS and PD scores at baseline were 10.3 (7.2) and 2.9 (3.9), respectively. In the PD-negative group, the mean (SD) GSS at baseline was 10.9 (11.2). The mean (SD) GS and PD score at baseline in the PD-positive group was 10.2 (6.0) and 3.7 (4.1), respectively. 63 (94.7%) and 30 (79.0%) patients had at least one joint with GSS or PD, respectively.

**Figure 2:**
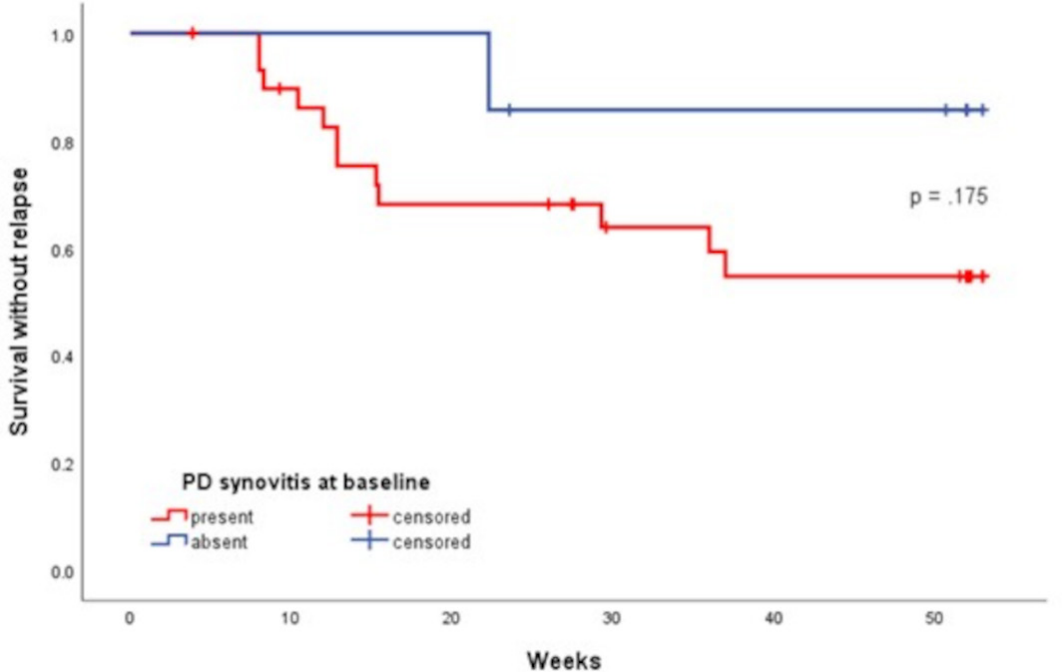
Kaplan-Meier analysis of relapses according to presence or absence of Power Doppler (PD) synovitis at baseline. Log-rank test yielded no statistically significant difference in the disease-free survival between the two groups (p=0.175).

Relapses between cessation of the bDMARD and week 16 (primary outcome) tended to be more common in patients with PD-positive synovitis at baseline, occurring in 9/30 patients (30.0%) in this group in comparison to 0/7 patients in the PD-negative group (p=0.160). One patient was excluded from the analysis because missing data precluded classification of disease activity status. Similar observations were made at weeks 24 (9/30 (30.0%) vs 1/7 (14.3%), p=0.647)) and 52 (12/30 (40.0%) vs 1/7 (14.3%), p=0.383)). The relapse-free survival of patients with and without PD-positive synovitis at baseline is depicted in figure 2 (p=0.175).

Furthermore, we investigated whether PD scores were higher at time of relapse than at the preceding visit: we observed a mean increase of 3.2 (±4.5, p=0.034) points. For relapsing patients, we recorded the PD score at relapse and the preceding visit; for non-relapsing patients, we calculated the mean PD score across all visits. The PD scores that preceded a relapse (mean 5.6 (±3.9), n=13) and those at the time of the relapse (mean 8.8 (±6.4), n=13) were significantly higher than the average PD scores of patients without a relapse (mean 2.0 (±1.5), n=24, p=0.003 and p=0.005, respectively). Explorative endpoints and Kaplan-Meier analysis of relapses according to presence or absence of at least one swollen or TJ at baseline are shown in online supplemental table 6 and figure 1.

## Discussion

The primary objective of this study was to investigate whether PD-positive synovitis predicted a relapse after stopping a biological agent in patients with RA in stringent clinical remission. While our data suggest that this hypothesis might be true, the study had to be terminated prematurely because of insufficient recruitment. All results should therefore be interpreted as exploratory. We observed a higher PD score at the time of relapse and preceding it as compared with phases in clinical remission, which might underline the use of ultrasound for a more accurate assessment of remission and relapse in patients with RA.

Several studies have investigated the potential value of imaging to predict a relapse during tapering or after discontinuation of bDMARDs.^9 11 17 27^ In a study of 77 patients with RA in sustained clinical remission, a higher PD synovitis score was associated with a lower likelihood of successful bDMARD tapering compared with a lower PD score.^11^ Similar findings have been reported by others.^15 16^ Some studies however have concluded that ultrasound provides no additional predictive value over clinical assessment alone for identifying flares in patients with RA discontinuing anti-tumour necrosis factor alpha (anti-TNFα) therapy.^7 12^

These heterogeneous results might be partially explained by differences in the applied remission and relapse criteria, time in remission before inclusion as well as different prevalence and level of PD-positive synovitis at baseline. Patients in Disease Activity Score 28 remission, for example, have higher residual inflammation than patients in sustained ACR/EULAR remission, potentially impacting the risk of relapse.^28^ Besides, ultrasound sensitivity for detecting synovial vascularisation increases, and PD signals are now detectable even in healthy individuals^29^; hence, a PD cut-off of one might be too low for relapse prediction in RA.

The main limitation of our study is inadequate patient recruitment, which resulted from a culmination of factors, including an overestimation of the willingness of patients with RA to stop their bDMARD therapy. This may also have introduced selection bias towards patients with milder disease, a more favourable disease course and higher expectations of maintaining remission. An alternative treatment strategy could have been to taper the drug, as performed in other studies.^11 14 16^ This would have presumably increased the sample size. A formal comparison between participants and non-participants was not feasible, as ethical and data protection regulations precluded the collection of data from individuals who declined participation. Consequently, the extent of potential non-response bias could not be assessed. However, investigators involved in patient recruitment observed that individuals with limited proficiency in German appeared less likely to participate, which may have introduced a form of selection bias. Other limitations include the lack of statistical adjustment for within-patient measurements for some secondary outcomes, the absence of data on inter-rater reliability of sonographers and the fact that only a 14-joint count was used for ultrasound assessment. This decision was made to improve the feasibility of the study, acknowledging that some patients with PD-positive synovitis in joints not included in the score might have been misclassified. Detailed information regarding the exact methotrexate dose and route of administration (subcutaneous vs oral) was not systematically collected and thus could not be explored.

In summary, while our trial failed to meet its primary endpoint, the numerically higher rate of relapses in patients with PD-positive synovitis as compared with those without, as well as promising results concerning selected secondary outcomes are hypothesis-generating, stimulating further research on the potential value of ultrasound for patient stratification and treatment decisions.

## Supplementary material

10.1136/rmdopen-2026-007003 online supplemental file 1

10.1136/rmdopen-2026-007003 online supplemental file 2
