# Supplementary material for: Ultrasound-based withdrawal of biologics in rheumatoid arthritis (RA-BioStop)
Source: RMD Open. 2026 Aug 7;12(3):e007003. doi: 10.1136/rmdopen-2026-007003 (PMC13475499; doi:10.1136/rmdopen-2026-007003)
Supplement: Supplementary file 1 [file rmdopen-12-3-s001.docx]

**Study protocol:**

**Ultrasound as biomarker for withdrawal of biological DMARDs in rheumatoid arthritis (RA-BioStop)**

**Short title: RA-BioStop**

**Design**: Prospective, multicentre, open study

# 1.: Abbrevations

| CRP | C-reactive protein |
| --- | --- |
| DMARD | disease modifying antirheumatic drug |
| EGA | evaluator’s global assessment of disease activity |
| ESR | erythrocyte sedimentation rate |
| EULAR | European League Against Rheumatism |
| i.v. | intravenous |
| p.o. | per os |
| PD | power Doppler |
| PGA | patient’s global assessment of disease activity |
| RA | rheumatoid arthritis |
| RF | rheumatoid factor |
| s.c. | subcutaneous |
| SDAI | simplified disease activity index calculated as follows: TJ + SJ + PGA + EGA + CRP |
| SJ | swollen joint |
| SPC | summary of product characteristics (=Fachinformation) |
| TJ | tender joint |
| TNF-alpha | tumour necrosis factor-alpha |

# 2.: Synopsis

| **Principal investigator**: | Dejaco Christian, MD, PhD, PD, MBA |
| --- | --- |

**Title**: Ultrasound as biomarker for withdrawal of biological DMARDs in rheumatoid arthritis (RA-BioStop)

**Short title**: RA-BioStop

| **Centers:** | Division of Rheumatology, Department of Medicine III, Medical University Vienna |
| --- | --- |
|  | Department of Internal Medicine II, Medical University Innsbruck |
|  | Department of Rheumatology, Medical University Graz |
| **Patients** | Consecutive RA patients treated with a combination of conventional synthetic disease modifying antirheumatic drugs (csDMARD) and a biological DMARD (bDMARD), who are in persistent clinical remission for at least 6 months |
| **Study design** | Prospective, Phase IV |
| **Objectives:** | Biological DMARDs may be stopped in RA patients treated with a combination of csDMARD and bDMARD, who are in persistent clinical remission. Ultrasound may predict a disease flare in patients stopping the bDMARD |
|  | **Primary objective**:  Active inflammation at the time of bDMARD withdrawal indicated by the presence of a PD-score ≥1 in at least one joint out of a sonographic 14-joint count predicts relapse rate at week 16. |
|  | **Secondary objectives:**   1. Active inflammation at the time of bDMARD withdrawal indicated by the presence of a PD-score ≥1 in at least one joint out of a sonographic 14-joint count predicts relapse rate at week 24. 2. Active inflammation at the time of bDMARD withdrawal indicated by the presence of a PD-score ≥1 in at least one joint out of a sonographic 14-joint count predicts relapse rate at week 52. 3. RA-patients have higher PD-scores at the time of a clinical flare compared to patients with maintained clinical remission. 4. An increment of PD-scores at follow-up compared to baseline visits precedes a clinical flare 5. PD-scores better predict a relapse than the presence of residual swollen joints 6. The higher the PD score at baseline, the more likely is a relapse at weeks 16, 24 and/or 52 7. Patients converting from a rheumatoid factor (RF) positive to a RF negative status are less likely to experience a relapse at weeks 16, 24 and 52 than patients remaining seropositive 8. Blood biomarkers predict the time to flare after bDMARD withdrawal 9. Blood biomarkers predict the time to re-achieve remission after flare and re-induction of bDMARD treatment 10. PD-scores and blood biomarkers at baseline predict radiographic progression at week 52 |
| **Intervention** | 1. Discontinuation of bDMARD, continuation of csDMARDs 2. 9 Study visits within 52 weeks including clinical, radiographic and laboratory routine assessments, ultrasound of 14 joints. 3. Collection of blood samples at baseline, after 2 weeks (study visit 2), after 12 weeks (study visit 5) and at time of a flare for the assessment of blood biomarkers |
| **Cohort size** | 110 patients |
| **Time plan** | Total duration of the study: 9 Years   1. Active phase for each patient: 12 months 2. 2. Recruitment: 8 years |
| **Inclusion criteria** | 1. Male or female patients ≥18 years and <90 years of age 2. Classification of RA according to the ACR-EULAR 2010 criteria 3. Clinical remission according to ACR-EULAR remission criteria at time of inclusion and remission or low disease activity according to SDAI in the preceding 6 months 4. Written informed consent 5. Current treatment with a csDMARD plus a stable dose and administration interval (for at least 6 months) of a bDMARD 6. No current corticosteroid treatment 7. Stable dose of NSAIDs |
| **Exclusion criteria** | 1. Current treatment with any investigational drug# 2. Current administration interval of the bDMARD of >11 weeks 3. Complete destruction of any joint to be investigated by sonography 4. Severe systemic (i.e. extraarticular) RA- manifestation 5. Begin of arthritis before age of 17 years 6. Planned surgery within the study period or history of surgery of any joint to be investigated clinically or by sonography 7. Current severe medical illness requiring hospitalization 8. Active infection or active malignancy 9. Pregnancy or lactation 10. Inability of the patient to follow the protocol 11. Current treatment with Rituximab (MabThera®) |

# 3.: Introduction

## 3.1.: There are insufficient data on the management of RA patients in clinical remission

Drug treatment of rheumatoid arthritis (RA) patients usually comprises disease modifying antirheumic drugs (DMARDs), corticosteroids and non-steroidal anti-inflammatory drugs [1]. The mainstay of RA treatment, however, is the application of DMARDs. Apart from conventional synthetic DMARDs (csDMARDs), most of which have been recognized as effective treatments for several years, biological agents (bDMARDs) including TNF-alpha inhibitors, the interleukin-6 receptor inhibitor tocilizumab, the CTLA-4 fusion protein abatacept and the anti-CD20 antibody rituximab have been established as an add-on therapy in RA patients refractory to csDMARD treatment [2]. The combination of csDMARDs and bDMARDs is highly effective as demonstrated by several clinical trials [3,4]. Particularly the number of patients achieving clinical remission increased from only 5-10% treated with csDMARDs to 20-30% of patients receiving the combination therapy [5].

Whereas numerous studies tried to identify the best treatment strategy to achieve remission in (new) active RA patients, little is known about the management of these patients once remission or low disease activity is achieved [6–8]. According to the new EULAR recommendations for the management of RA, cessation of bDMARDs may be considered in patients with persistent remission after having tapered glucocorticoids [4]. In addition, the expert committee felt that csDMARDs should not be discontinued because one randomized controlled trial on stopping csDMARDs in patients in remission showed that half as many patients who maintained their csDMARDs flared as compared with those who stopped them [9]. Moreover, remission was much harder to re-achieve after stopping csDMARDs [10].

Discontinuation of biological agents in patients with established RA was studied in the ‘Remission Induction by Remicade in RA’, the PRESERVE and the HONOR trials demonstrating relapses in 45% to 57% of patients after anti-TNF alpha withdrawal [11–13]. OPTIMA and the BEST study (investigating initial combination therapy in early RA) reported that remission or low disease activity persisted in the majority of patients, once remission was achieved and anti-TNF-alpha agents were discontinued [12,14]. In the ACT-RAY study, relapses were common upon cessation of tocilizumab; however, all patients responded well to re-treatment with tocilizumab and no relevant radiographic damage was observed [15]. In a Japanese multicenter open study, 41% of patients in whom abatacept was discontinued, remained in remission until the end of the observational period of 52 weeks [16].

Predictors of a good outcome after bDMARD discontinuation have rarely been studied. One study suggested that patients with a profound and persistent response to anti-TNF alpha therapy had a higher likelihood to maintain response with csDMARDs alone than patients with an incomplete response [11]. Whether the absence of inflammation as determined by ultrasound is also a valuable predictor of persistent bDMARD-free remission remains elusive so far.

## 3.2.: Value of sonography for the detection of joint inflammation in rheumatoid arthritis

Musculoskeletal sonography is increasingly used for diagnosis and follow-up of RA patients [17]. Despite clear evidence supporting the diagnostic impact of ultrasound for arthritis, the new 2010 ACR/EULAR classification criteria for RA did not include sonographic findings as an obligatory parameter [18]. The authors of these criteria as well as the new EULAR recommendations for the use of imaging in RA emphasized the possibility that sonography may be used to confirm the clinical finding of an ‘involved’ joint in patients with at least one joint with definite clinical synovitis (not better explained by another disease) [19]. One recent study suggested that ultrasound investigations may result in a considerable shift from monoarthritis to oligoarthritis or polyarthritis in patients with very early arthritis; and the addition of sonographic findings to the 2010 ACR/EULAR criteria increased the number of patients also fulfilling the 1987 ACR criteria after an 18 months follow-up period [20].

Regarding the follow-up of patients with established RA, EULAR stressed the fact that ultrasound is superior to clinical examination for the detection of joint inflammation and should thus be considered for a more accurate assessment of inflammation [19]. Power Doppler (PD)-signals were observed in up to 60% of RA patients in clinical remission and conversely, sonographic signs of inflammation were absent in 20% of RA cases despite increased disease activity scores [21–25]. When sonographic data were added to SDAI clinical composite index, an additional 32.3% of patients could be classified as having high disease activity at the screening visit of a randomized controlled trial on etanercept and methotrexate in patients with moderate RA [26].

Among available clinical (composite-score based) remission criteria it seems that the ACR/EULAR criteria (and particularly the SDAI) best reflect the absence of ultrasound verified inflammation [27,28].

Most ultrasound studies in RA reported a good to excellent inter- and intraobserver reliability of sonography results [17,29] whereas clinical joint counts are limited by a high interobserver variance and unsatisfactory sensitivity and specificity to detect joint inflammation [30,31]. Moreover, ultrasound scores revealed a good sensitivity to change in follow-up studies of RA patients treated with corticosteroids or biological agents [32–37].

## 3.3.: Prognostic value of baseline ultrasound evaluation in rheumatoid arthritis

Treatment decisions in rheumatology are usually based on prognostic factors predicting structural outcomes as well as clinical and functional treatment success. In RA, high disease activity state, autoantibody positivity [rheumatoid factor (RF) and/or antibodies to citrullinated proteins (ACPA)] and the early presence of joint damage are associated with a high risk of rapid radiologic damage [38,39]. Current EULAR recommendations for management of RA suggest a more intensive treatment strategy in patients with poor prognostic factors compared to cases without these factors [4]. Baseline ultrasound results may extend clinical risk assessment in patients with established RA predicting treatment responses as well as structural progression as outlined below.

### 3.3.1.: Ultrasonography as predictor of treatment response

RA patients with a high number of PD-signals at baseline were found to have a worse response to biological treatments, and the grade of ultrasound-verified inflammation predicted the maintenance of anti-TNF-α therapy after one year [40,41]. Both studies, however, are compromised by small sample size stressing the need for additional studies investigating the role of sonography as a predictor of response in RA patients treated with biological agents.

### 3.3.2.: Ultrasonography as prognostic factor for structural deterioration

Several studies demonstrated that ultrasound-verified inflammation is linked to the occurrence of erosive lesions as verified by X-ray, MR or CT [42–44]. This association was strongest at the level of individual joints as compared to the patient- or group level. An even higher association between sonography and radiographic progression was observed taking time integrated ultrasound-results (including baseline and follow-up examinations) into account [45].

## 3.4.: Relevance of ultrasound verified subclinical inflammation in rheumatoid arthritis

### 3.4.1.: Ultrasonography as predictor of structural deterioration and clinical relapse in patients in clinical remission

It is well established that patients in clinical remission have better clinical and structural outcomes than patients with active disease [5]. Radiographic progression, however, is not abrogated in all patients in clinical remission or low disease activity [5] and this fact may be explained by the presence of subclinical inflammation, as observed in a considerable proportion of patients by PD ultrasound [23,46]. Ultrasound assessment of patients in clinical remission has also been of interest in the context of flare prediction: RA patients with persistent clinical remission having at least one PD-signal in a 44-joint count were at a 12-fold increased risk for short term clinical relapse compared to patients without PD-signals [21]. A similar result was obtained in another follow-up study comparing patients with early and long-standing RA [24]. In a study by Saleem et al, the predictive value of ultrasound findings was compared with clinical remission criteria concerning the occurrence of future flares. The authors reported that the presence of PD-signals was the strongest predictor of a relapse whereas clinical remission criteria were less useful for this purpose [47].

Recent data, however, questioned the value of low-grade PD signals as indicators of active synovitis reporting that only high- but not low-grade PD signals are relevant for clinical and functional outcomes in RA patients in clinical remission [48]. In addition, although correlation was demonstrated between histology and sonographic synovitis scores in patients in clinical remission, this was not significant [49]

### 3.4.2.: Ultrasonography as predictor of relapse after bDMARD withdrawal

Only few studies so far have investigated whether ultrasound findings may support the decision to withdraw bDMARD therapy [22,50,51]. Saleem et al. reported that early instigation of the bDMARD was associated with persistent remission whereas ultrasound results did not predict successful anti-TNFα cessation. In contrast, Iwamoto et al found high relapse rates in patients with high gray-scale and PD scores at time of discontinuation of the biological agent. Both studies, however, were limited by small sample size and the inclusion of patients with DAS-28 rather than ACR/EULAR remission criteria. Given that current EULAR recommendations discourage the cessation of bDMARDs in patients not fulfilling the ACR/EULAR remission criteria [4], the clinical value of these studies is limited. Another observational study of RA patients in remission investigated the value of sonography to predict successful tapering of biological agents [51]. The authors reported that the PD score at baseline was the strongest predictor for biological tapering failure; however, the study was limited by the use of inadequate remission criteria, variable tapering regimens and pooled analysis of patients with different biological substances.

## 3.5.: Autoantibodies in RA

RF and ACPA are well-established biomarkers in RA. They are used for diagnostic purposes, have been included in current classification criteria and serve for prognostic evaluation of RA patients [18,52]. ‘Seropositive’ patients are considered to have more aggressive disease and are at a higher risk of structural progression than patients without these autoantibodies [53,54]. Recent data indicate, however, that autoantibodies, particularly the RF, may not persist in all RA patients and that ‘seropositive’ patients may change into a ‘seronegative’ status during follow-up [55].

The RF can be detected in 60-80% of RA patients as well as in 5% of healthy individuals. A negative result does therefore not exclude the disease and a positive result is insufficient to confirm a diagnosis of RA [56].

The role of RF in the pathogenesis of RA is elusive so far. It has been described that RF-specific B cells may capture and present self-antigens to T cells by immune complex uptake. RF, after building immune complexes with autologous IgG [57], activates complement, including C5a, which subsequently binds to C5a receptors on macrophages leading to their activation. This contributes to the inflammatory cascade in RA finally leading to localized tissue damage [58].

The observation that the RF but not ACPA is linked with disease activity may partially be explained by the facts that RF is an IgM isotype whereas ACPA are mostly IgGs. Different Ig isotypes have specific effects on the immune response [59] and IgM for example, may activate complement to a greater extent than IgG [60]. This can boost the inflammatory response via inflammatory complement breakdown products and/or complement-receptor mediated macrophage activation [61]. In contrast, ACPA might preferentially activate inhibitory Fcγ-receptors mitigating the inflammatory response [62].

Apart from the different Ig isotypes of RF and ACPAs, the activation of cells producing these autoantibodies may have direct effects on the pathogenesis of RA. ACPAs are the products of long-lived memory plasma cells [63], which are less tightly linked to the inflammatory disease process [64]. The RF may be produced at least partly by a subset of B lymphocytes, namely B-1 cells [65], which have a clearly higher plasticity than plasma cells, and are therefore more closely linked to inflammation and clinical disease activity.

As mentioned above, RF and ACPA levels may not be constant in RA patients. Particularly in patients with an improvement of disease activity, the RF usually declines faster, to a larger extent and in a greater number of RA patients than ACPAs [55]. Whether these changes have any relevance for the prediction of the clinical outcome of patients discontinuing bDMARDs has not been investigated so far.

# 4.: Summary

There is insufficient data on the management of RA patients treated with biologic agents in clinical remission. Current EULAR recommendations for the management of RA suggest that cessation of bDMARDs may be considered in patients with persistent remission after having tapered glucocorticoids [4]. Predictors for the successful withdrawal of biological agents, however, are scarce.

Sonography is increasingly used as a prognostic marker in RA. Subclinical inflammation may be of high relevance for future clinical and structural outcomes. An increased risk for short-term clinical relapses for example, may occur in patients with intraarticular PD-signals. The RF may predict clinical and radiographic outcomes of RA patients. Successfully treatment of RA result in a reduction of RF blood levels.

Whether ultrasonography and/or blood biomarkers such as RF might predict a relapse after discontinuation of biological agents remains elusive so far.

# 5.: Rationale

bDMARDs are effective therapies for RA. These substances, however, are expensive and their long-term use may be associated with an increased risk of infections and other side effects. Whether RA patients in persistent clinical remission require continued application of bDMARDs is insufficiently known.

According to the 2013 EULAR recommendations for the management of RA discontinuation of bDMARDs may be considered in patients with persistent clinical remission [4].

Ultrasound is increasingly used as a predictor in RA [17]. Previous studies indicated that ultrasound-determined active inflammation predicts future clinical deterioration and progression of radiological damage despite clinical remission or low disease activity, whereas patients without sonographic signs of disease activity had a more favorable outcome [21,23,24].

The RF has been shown to predict disease progression of RA; however, the relevance of this factor as well as other blood biomarkers for the prediction of a relapse in patients stopping bDMARDs has not been investigated so far.

We hypothesize that sonography and blood biomarkers may be useful to predict a disease flare after discontinuation of bDMARDs in RA patients.

# 6.: Objectives

bDMARDs may be stopped in RA patients treated with a combination of csDMARD plus bDMARDs who are in persistent clinical remission. Ultrasound may be useful to predict a disease flare in patients stopping bDMARD therapy.

## 6.1.: Primary objective:

Active inflammation at the time of bDMARD withdrawal indicated by the presence of a PD-score ≥1 in at least one joint out of a sonographic 14-joint count predicts relapse rate at week 16.

## 6.2.: Secondary objectives:

1. Active inflammation at the time of bDMARD withdrawal indicated by the presence of a PD-score ≥1 in at least one joint out of a sonographic 14-joint count predicts relapse rate at week 24.

2. Active inflammation at the time of bDMARD withdrawal indicated by the presence of a PD-score ≥1 in at least one joint out of a sonographic 14-joint count predicts relapse rate at week 52.

3. RA-patients have higher PD-scores at the time of a clinical flare compared to patients with maintained clinical remission.

4. An increment of PD-scores at follow-up compared to baseline visits precedes a clinical flare

5. PD-scores better predict a relapse at week 16, 24 and 52 than the presence of residual swollen joints

6. The higher the PD score at baseline, the more likely is a relapse at weeks 16, 24 and/or 52

7. Patients converting from a RF positive to a RF negative status are less likely to experience a relapse at weeks 16, 24 and 52 than patients remaining seropositive

8. Blood biomarkers predict the time to flare after bDMARD withdrawal

9. Blood biomarkers predict the time to re-achieve remission after flare and re-induction of biological treatment.

10. PD-scores and blood biomarkers at baseline predict radiographic progression (according to the van der Heijde/Sharp method) [66] at week 52

# 7.: Methods, study design

This is a prospective, multicenter, clinical phase IV trial.

Consecutive patients with known RA treated with a combination of csDMARD plus bDMARDs and persistent (=at least 6 months) clinical remission as defined below will be included in the study csDMARDs are the following: Ebetrexat®, Methotrexat® (methotrexate), 2.5mg-40mg once a week, p.o. or s.c.; Salazopyrin® (sulfasalzine), 500mg-3000mg daily p.o.; Imurek®, Immunoprin®, Azathioprin® (azathioprine), 50mg-200mg daily p.o.; Arava® (leflunomide), 10mg-30mg daily p.o.; Resochin® (chloroquine), 250mg daily p.o.; Quensyl (hydroxychloroquine), 200-400mg daily p.o.; Sandimmun® (cyclosporine), 50-300mg daily p.o.

bDMARDs are the following: Infliximab (Remicade®, Remisima®, Inflektra®), 3-10mg/kg body weight, administered every 6-8 weeks i.v.; Etanercept (Enbrel®), 25mg twice a week or 50mg once a week s.c.; Adalimumab (Humira®), 40mg every other week s.c., Certolizumab pegol (Cimzia®), 200mg every other week s.c., Golimumab (Simponi®), 50mg every month s.c.; Tocilizumab (Roactemra®), 8mg/kg body weight, administered every 4 weeks or 162mg once a week s.c., Abatacept (Orencia®), 500-1000mg administered every 4 weeks i.v. or 125mg once a week s.c.

A reduction of the bDMARD dose or an extension of the infusion/injection interval can be performed in patients attaining clinical remission [3,4]. The biological agent is stopped after inclusion into the study (i.e. it will not be administered at baseline or thereafter) and clinical and ultrasound assessments are scheduled as outlined below.

Patients treated with Rituximab (MabThera®) (at the time of screening) are excluded from this study for the following reasons: 1) the administration interval and the immunosuppressive effects of this drug are fundamentally different from other biological agents. 2) There is considerable heterogeneity regarding the use of Rituximab doses (500mg vs. 1000mg for maintenance therapy) and administration intervals (administration every 6-month vs. administration on demand in case of increasing disease activity) in clinical practice [67,68].

## 7.1.: Inclusion criteria

- Male or female patient ≥18 years and <90 years of age
- Classification of RA according to the ACR-EULAR 2010 criteria
- Clinical remission according to ACR-EULAR remission criteria at time of inclusion and remission or low disease activity according to SDAI in the preceding 6 months (documented at ≥1 visit) [5]
- Written informed consent
- Current treatment with a single csDMARD or a combination of csDMARDs plus a stable dose and administration interval (for the last 6 months) of a bDMARD
- No current systemic corticosteroid treatment (stopped for at least 4 weeks), no corticosteroid injection within 4 weeks
- Stable dose of NSAIDs for at least 1 week

## 7.2.: Exclusion criteria

- Current treatment with any investigational drug
- Current administration interval of the bDMARD of >11 weeks
- Complete (clinically evident) destruction of any joint to be investigated by sonography as judged by the physician
- Current RA-related vasculitis or other active systemic (i.e. extraarticular) RA- manifestation with the exception of rheumatoid nodules
- Initial arthritis manifestations before the age of 17 years
- Planned surgery within the study period or history of surgery of any of the joints to be investigated clinically or by sonography
- Current severe medical illness requiring hospitalization
- Active infection or active malignancy at screening or infection during the past 4 weeks requiring (even temporary) discontinuation of the bDMARD
- Pregnancy or lactation
- Inability of the patient to follow the protocol
- Current treatment with Rituximab (MabThera®)

## 7.3.: Investigations and interventions

### 7.3.1.: General aspects

Consecutive RA patients of the outpatient clinics of the participating centers fulfilling inclusion and exclusion criteria will be recruited. Written informed consent will be obtained. All investigations will be performed within the study centers. Baseline visit will be performed at the day normally scheduled for the bDMARD administration. Screening and baseline visit may be performed at the same or at two separate days (in the latter case the period between screening and baseline is 6 weeks at maximum). At the screening visit, patients will sign the informed consent form and the physician will check in- and exclusion criteria. At the baseline visit, in- and exclusion criteria will be assessed again. After inclusion into the study, the biologic drug will be discontinued (i.e. the bDMARD will not be administered at baseline or thereafter) in all consenting participants while treatment with csDMARDs will be continued. Study visit 2, 3, 4, 5, 6, 7, 8 and 9 will be performed at weeks 2, 4, 8, 12, 16, 24, 36 and 52 (+/- 3 days)respectively (see flow chart below). Unscheduled visits may be performed at any time at the discretion of the treating physician, particularly in case of adverse events or suspected relapse.

A clinical relapse is defined by a change of disease status from ACR/EULAR remission to moderately active disease (as defined by a SDAI > 11). Patients experiencing a relapse are classified as discontinuation-failures and terminate the active phase of the study. These patients are treated according to routine guidelines for RA (e.g. by re-administration of the biologic agent) but are still followed up until week 52 including clinical, laboratory and ultrasound assessments.

## 7.4.: Clinical and routine laboratory assessments

At each study visit (including screening) patients undergo full clinical history and clinical assessment including tender joint (TJ) count (28 joints), swollen joint (SJ) count (28 joints), global assessment of disease activity by the patient (PGA) and physician (EGA), pain assessment by the patient (pt-VAS). Global and pain assessments are recorded on a 100mm visual analogue scale with 0=best, 100= worst. Routine blood investigations including ESR, CRP, blood count, RF, anti-CCP, liver and renal functional tests are performed at each visit. A pregnancy test (urine test) will be conducted before, and monthly upon the inclusion into the study.

## 7.5.: Ultrasound investigations

At each visit, patients will undergo an ultrasound examination of 14 joints [s, 2nd and 3th metacarpo-phalangeal (MCP), 2nd and 3th proximal interphalangeal (PIP), 2nd and 5th metatarso-phalangeal (MTP)] at palmar and dorsal sites by one independent investigator at each center, blinded to all clinical data. This 14 joint ultrasound count is based on a modification of the German U7 score toward the bilateral (instead of unilateral) joint assessment, because we deemed that the identification of the clinically dominant site (as stipulated by the original U7 score) may not necessarily be reliable in patients in clinical remission [69–71].

The investigator performing ultrasound is not involved in any other part of the study, clinical decisions or routine examinations. Patients are asked not to talk about their complaints or the result of the clinical visit.

## 7.6.: Radiographic imaging and scoring

Radiographs of the hands and feet will be performed routinely at baseline unless recent radiographs (less than 6 months ago) are available. A radiographic follow-up examination will performed after 12 months, as part of clinical routine [19]. Radiographs will be scored according to the van der Heijde/Sharp method [66]. Radiographs will be scored pairwise with knowledge of the temporal sequence, and results will be documented by joint and by type of involvement (erosions, JSN) into a radiographic dataset, which is integrated into the routine dataset.

## 7.7.: Blood and urine biomarkers

For this part of the study, patients will sign a separate informed consent form. For the analysis of biomarkers, we will obtain blood (as detailed below) and urine (10ml at each of the following visits) samples at baseline, after 2 weeks (study visit 2), after 12 weeks (study visit 5) and at time of a flare. In addition to the routine blood samples (20ml), we will collect 43ml blood at the baseline visit (thus totalling 63ml) and 40ml at visit 2 and 5 as well as at the flare visit (total 60ml). DNA isolation will be performed at baseline only. Specifically, we will collect the following samples:

- 1x Serum tube containing separator gel (GBO Vacuette Serum 8mL, #455071) (all 4 visit as stated above)
- 3x K2EDTA tube containing separator gel (GBO Vacuette K2EDTA 8mL, #455021) (all 4 visit as stated above)
- 1x K3EDTA tube (GBO Vacuette K3EDTA 8mL #454217) (all 4 visit as stated above)
- 1x Urine (GBO Vacuette Z Urine without additive 10ml #455007) (all 4 visit as stated above)
- 1x K3EDTA tube (GBO Vacuette K3EDTA 3mL #454217) (at baseline only)

We will be perform flow cytometry for the analysis of T- and B-cell subsets within 24h of blood withdrawal in the laboratories of the Department of Rheumatology and Immunology, Medical University Graz, the Division of Rheumatology, Department of Internal Medicine III, Medical University Vienna and the Department of Internal Medicine VI, Innsbruck Medical University, as appropriate. All other Materials will processed and stored at the Department of Rheumatology and Immunology of the Medical University of Graz as well as at the “Biobank” of the Medical Universities of Vienna and Innsbruck until further assessments. All biomarker and genetic studies will be conducted at the Medical University Vienna, Division of Rheumatology, Department of Internal Medicine III in cooperation with the Department of Medical and Chemical Laboratory Diagnostics, after termination of the study.

Specifically, we plan to conduct the following analyses:

### 7.7.1.: Markers of disease activity and bone and cartilage turnover

Serum and urine samples will be analysed using ELISA technique for the concentration of the following markers of inflammation, cartilage and bone damage and repair: a) inflammation: sAA, IL-1, IL-6, TNF, IL-17, IFNα, IFNγ, MMP-3, MMP-9; b) cartilage damage and repair: N-terminal propeptide of type IIA collagen (PIIANP) cartilage oligomeric protein (COMP), C-terminal crosslinking telopeptide of type II collagen (CTX-2), collagen helical peptideHelix-2;, and c) bone destruction and repair: osteocalcin, N-terminal propeptide of type I collagen (PINP) C-terminal crosslinking telopeptide of type I collagen (CTX-1), C-terminal crosslinking telopeptide of type I collagen generated by matrix metalloproteinases (ICTP), cathepsin K; soluble receptor activator of NFκB ligand (RANKL), osteoprotegerin (OPG). Most of these markers have been shown to reliably reflect inflammation/the inhibition of inflammation and/or joint destruction in RA and to be sensitive to change with anti-inflammatory therapy.

### 7.7.2.: Cellular analyses of the immune system

PBMC samples will be collected for flow cytometric analysis of leukocyte subsets including CD3, CD4, CD8, CD19, CD21, CD24, CD25, CD27, CD28, CD38, CD86, CD127, CD183, CD194 (CCR4), CD196 (CCR6), CD197 (CCR7), CD45RA, IgM. Staining of the cells and flow cytometry will be performed using a standard protocol in the laboratories of the Department of Rheumatology and Immunology, Medical University Graz, the Division of Rheumatology, Department of Internal Medicine III, Medical University Vienna and the Department of Internal Medicine VI, Innsbruck Medical University, as appropriate.

### 7.7.3.: Gene expression analyses

Gene expression profiling of RNA obtained from whole blood will be performed by microarray technique (Affymetrix, GeneChipPrimeView Human Gene Expression array) to identify up- or downregulation of individual genes associated with disease activity after discontinuation of the biological agents and the predictive value of these markers with future relapses. Microarray analyses are well established at the Department of Medical and Chemical Laboratory Diagnostics of the Medical University of Vienna, where the analyses will be performed.

### 7.7.4.: Genotyping

Genotyping will be performed on baseline samples only using the Affymetrix Genome-Wide Human SNP Array 6.0. While a number of polymorphisms at different loci, especially HLA-DRB1 (shared epitope), PTPN22, TRAF1, C5, and STAT4, have been shown to be associated with RA, a broader analysis will be needed, since it is currently unknown which other genes may be of potential predictive value for flares. These analyses will be performed the at the Division of Rheumatology, Department of Internal Medicine III, Medical University Vienna in cooperation with the Department of Medical and Chemical Laboratory Diagnostics of the Medical University of Vienna.

## 7.8.: Definition of a serological change of the Rheumatoid factor

The RF has the potential to change under therapy [55]. The usual cut-off value for a positive RF test result is 14-20 IU/ml (depending on the test system used); however, this cut-off results in a moderate specificity for RA only. It has been shown that titres above 50 IU/ml have a much higher specificity for the diagnosis of RA [72,73]. To show a clear signal of immunological change, we choose a cut-off value of > 50 IU/ml as an absolute criterion for RF positivity, and we define a decline of RF levels below this cut-off and a relative change of at least 50% as seroconversion.

## 7.9.: Visit schedule and assessments

## 7.10.: Ultrasound protocol

Patients are examined in sitting position with hands or feet positioned on the examination table in a darkened room in which the temperature is held constant at 20°C. Scan images are recorded on the hard drive of the US unit. A high-end US device will be used in each center. The PD-gain will be optimized by increasing gain until random noise appears and then reduced just enough to suppress the noise particularly at the bony cortex. Motion artifacts are minimized by the use of a wall filter (medium level) as well as by appropriate positioning of the patient and investigator. Mirror artifacts are recognized when the true image as well as the mirror (usually the bony cortex) and mirror image are all in the same image. The PD box will be extended to include the top of the image to avoid reverberation artifacts. Both B-mode and PD-examinations are performed; transverse and longitudinal scans of the dorsal and palmar aspect of each included joint are acquired according to the published guidelines [74]. MCP and PIP joints are examined in the extended position; wrists and MTP joints in neutral position.

Grey-scale synovitis (GSS) is identified by B-mode imaging in each joint as abnormal, intraarticular, hypoechoic, poorly displacable material (corresponding to synovial hypertrophy) and/or intraarticular anechoic displacable material (corresponding to synovial effusion), respectively, and are used as a combined measure as proposed previously [71]. Semiquantitative scoring will be performed as following: GSS is subjectively graded from 0 to 3 in which 0 represents no GSS, 1=minimal GSS; 2=moderate GSS and 3=extensive GSS. PD-signals are also semiquantitatively graded on a scale from 0–3, in which 0 represents no PD-signal, 1=up to three single or two confluent vessels, 2=less than half of the synovial area and 3=half or more of the synovial area covered by PD-signals. PD-signals are counted in the systolic phase only. Scores are expressed per joint (possible range 0-6), and a total score is calculated by the summation of all joint scores (global ultrasound score with a possible range 0-84, GSS score with a possible range from 0-42 and a PD-score with a possible range from 0-42). Ultrasound examinations will be performed by one investigator out of the US personnel available at each site. For the purpose of acquisition reliability assessment, two investigators of the Medical University Graz will perform sonography in 20% of patients. Reading reliability will be tested by blinded scoring of stored loops of anonymized standard longitudinal scans acquired from 20% of patients. With this method, the reproducibility of ultrasound findings between the investigators of the different study centers will be evaluated. Intraobserver variability will be tested by one investigator at each site in 20% of patients by (1) blinded scoring of the stored loops >1 week apart and (2) by comparison of ultrasound results between screening and baseline visit (in case visits are > 1 day and <10 days apart).

## 7.11.: Allowed and prohibited medications

Analgetics are allowed during the study at the discretion of the treating physician (for example to treat concomitant osteoarthritis). All drugs used to treat concomitant medical conditions (e.g. hypertension) are allowed during the study. NSAIDs are allowed but should be taken at a stable dose as described above. Intraarticular injections (corticosteroids, local anestetics and/or hyaluronic acid) are also allowed (e.g. due to inflammatory arthritis) after baseline visit but only at joints that are not investigated by sonography and are not included in the 28-joint count [75].

The prescription of prohibited medications leads to the exclusion of the patient from the study. Oral, intramuscular and intravenous corticosteroids are prohibited during the active phase of the study. For RA patients in clinical remission, the prescription of corticosteroids is usually not indicated; in case corticosteroids are prescribed for 7 days or more due to other reasons (e.g. due to exacerbation of chronic obstructive pulmonary disease) patients are excluded from the study. New immunosuppressive drugs (e.g. for other indications) and/or chemotherapeutic agents are not allowed during the active phase of the study. In addition, dose of csDMARD treatment should not be changed during the active phase of the study. Rituximab or other biological agents (also if administered for other reasons than RA) as well as targeted synthetic DMARDs are not allowed during the study [76].

## 7.12.: Adverse Events

### 7.12.1.: Definition of an Adverse Event (AE)

Any untoward medical occurrence in a patient or clinical investigation subject administered a pharmaceutical product and which does not necessarily have a causal relationship with this treatment. An adverse event (AE) can therefore be any unfavourable and unintended sign (including an abnormal laboratory finding), symptom, or disease temporally associated with the use of a medicinal (investigational) product, whether or not related to the medicinal (investigational) product (see note for guidance on Clinical Safety Data Management: Definitions and Standards for Expedited Reporting).

### 7.12.2.: Serious Adverse Event (SAE), Serious Adverse Drug Reaction (Serious ADR)

Any untoward medical occurrence that at any dose:

- results in death,
- is life-threatening,
- requires inpatient hospitalisation or prolongation of existing hospitalisation,
- results in persistent or significant disability/incapacity,
- is a congenital anomaly/birth defect

### 7.12.3.: Suspected unexpected serious adverse drug reactions (SUSARs)

Suspected Unexpected Serious Adverse Drug Reactions (SUSARs) are side effects (probably or definitely connected with the investigational product), the nature or severity of which are inconsistent with the information available about the product. Information about the trial product contained in the SPC should be used to verify if the adverse reaction has been previously described.

## 7.13.: Safety reporting

### 7.13.1.: Role of the investigator for safety reporting

The investigator is responsible for reporting AEs, SAEs and SUSARs to the sponsor.

All serious adverse events (SAEs) should be reported immediately to the sponsor except for those SAEs that the protocol or other document (e.g., Investigator's Brochure) identifies as not needing immediate reporting. The immediate reports should be followed promptly by detailed, written reports. The immediate and follow-up reports should identify subjects by unique code numbers assigned to the trial subjects rather than by the subjects’ names, personal identification numbers, and/or addresses. The investigator should also comply with the applicable regulatory requirement(s) related to the reporting of unexpected serious adverse drug reactions to the regulatory authority/ies) and the IRB/IEC.

Adverse events and/or laboratory abnormalities identified in the protocol as critical to safety evaluations should be reported to the sponsor according to the reporting requirements and within the time periods specified by the sponsor in the protocol.

For reported deaths, the investigator should supply the sponsor and the IRB/IEC with any additional requested information (e.g., autopsy reports and terminal medical reports).

### 7.13.2.: Role of the sponsor

The sponsor should keep records of all AEs reported by the investigators. These records should be forwarded upon request to the competent authority of the region where the Clinical Trial is performed. In case of death or immediate danger of life caused by a SUSAR the competent authorities and Ethics Committee(s) concerned should be informed by the sponsor within 7 days after the event becomes known to the sponsor. Additional information should be given within further 8 days. All other SUSAR (“requires inpatient hospitalisation or prolongation of existing hospitalisation, results in persistent or significant disability/incapacity, or is a congenital anomaly/birth defect) should be reported as soon as possible, at latest within 15 days after the event becomes known to the sponsor, to the competent authorities and Ethics Committee(s) concerned.

Information on SUSARs should be forwarded to all other investigators of the same clinical trial by the sponsor. Once yearly during the trial, a safety report and a listing of all SUSARs that occurred during the trial should be forwarded to the competent authorities and to the Ethics Committees concerned by the sponsor.

As this study is an Investigator-Initiated-Study (IIS) and the sponsor is the principal investigator of the study all the duties concerning the documentation and reporting will be performed by the principal investigator.

## 7.14.: Settings

A high-end ultrasound device is available at each study centre. All ultrasound investigators have several years of clinical and research experience and were involved as teachers in international musculoskeletal ultrasound courses.

## 7.15.: Premature Termination

### 7.15.1.: Drop-out

Drop-out of single study participants may be due to

- withdrawal of informed consent
- intolerable adverse events
- violation of the study protocol
- emergence of an exclusion criterion
- emergence of concomitant disease
- pregnancy
- any other circumstance that would endanger the health of the study participant with continued study participation

### 7.15.2.: Discontinuation of the study

The investigator may discontinue the study anytime in the interest of the study participants if severe adverse events or other unforeseen problems occur.

Ethics Committees concerned and competent authorities should be informed about premature discontinuation and its reason within 15 days.

### 7.15.3.: Changes of the study protocol

Any amendment of the protocol must be announced to the ethics committee and the competent authority. Substantial amendments may be implemented only after approval of the ethics committee and competent authority, respectively.

## 7.16.: Monitoring

Monitoring will be performed according to the monitoring plan of this study. The investigator will cooperate with the monitor and provide unrestricted access to source data and all other study materials. The investigator will keep detailed records in the patient charts on study visits, distribution of study drugs, test results, concomitant diseases, and adverse events. The monitor will regularly verify entries on the CRFs with source data and check compliance with the study protocol and continuous data entry. The monitor will handle all data confidentially to ensure integrity and privacy of the study participants.

## 7.17.: Statistics

### 7.17.1.: Power analysis

In a previous, similar study a flare after cessation of anti-TNF therapy was reported in 60% of cases [22]. Assuming that 40% of the relapsing patients after bDMARD discontinuation have PD-signals at baseline compared to 10% without a relapse, a power of 90%, an alpha of 0.05 and an estimated drop-out rate of 10%, a total of 110 patients are required for this trial. Power calculation was performed assuming unequal group size (ratio 1.5) and using Fisher’s exact test.

A difference of 30% between the groups was chosen on a clinical basis because the investigators considered a smaller difference to have insufficient clinical relevance.

### 7.17.2.: Statistical analyses

Descriptive statistics, Fisher’s exact test, ROC-curve analysis, logistic regression analysis and survival analysis will be performed. For the primary hypothesis (active inflammation at the time of bDMARD withdrawal predicts relapse at week 16) Fisher’s exact test will be used. Logistic regression analysis (with adjustments for disease duration, erosive disease, RF, number of previous DMARDs, duration of remission before bDMARD withdrawal, bDMARD dose and administration interval before withdrawal, period of treatment with (if any) reduced bDMARD dose or extended bDMARD administration interval, duration of bDMARD treatment at the time of drug withdrawal and disease duration at the time of bDMARD initiation) will also be applied. The same methods will be applied for secondary hypothesis 1,2, 5 and 7 (for the latter using RF conversion from a positive to negative status as the predictor of primary interest). ROC-curve analysis is performed to search for a possible cut-off for ultrasound graded inflammation as a relapse predictor (secondary hypothesis 6). Survival analysis is performed to investigate the predictive value of sonography for the time in remission after withdrawal of the bDMARD agent. A Cox-regression model with PD-scores as a time depended covariate is conducted to test secondary hypothesis 4. For secondary hypothesis 3 the Wilcoxon test is used. Exploratory analyses are used for secondary hypotheses 8-10. If patients terminate the study for other reasons (e.g. due to prohibited medication) the patient will be excluded from final analysis (per protocol analysis). Interobserver and intraobserver variability of ultrasound investigations are tested using intra-class correlation coefficient (ICC). A p-value <0.05 will be considered significant. All analyses will be done with SPSS, Version 23.0 (Chicago, IL, USA).

# 8.: Data management

All data acquired during the study will be made anonymous for further analyses. Written informed consent is obtained from each patient and signed forms will be filed for 15 years at the Medical Universities of Graz, Vienna and Innsbruck. Each patient will receive a copy of the informed consent form

# 9.: Ethical considerations

Although bDMARDs are effective for treatment of RA, their long-term use is associated with increased risk of side effects including hypersensitivity reactions and/or infections, particularly tuberculosis. Besides, these treatments are among the most costly substances available to date. Whether patients in clinical remission require continuous treatment with bDMARDs has not been clarified so far. It was reported that 40-55% of patients may discontinue anti-bDMARD treatment without relapse, however, reliable biomarkers predicting a flare or persisting remission have not been identified so far [11–13]. Sonography is a promising biomarker in RA and the absence of inflammation as verified by ultrasound may support the clinical decision to stop the biological agent.

We anticipate that the conclusions drawn from this study improve future management of RA patients in remission treated with csDMARDs and bDMARDs. The individual patient may benefit from regular ultrasound monitoring and, in case of successful withdrawal of the biologic agent, from a reduced risk of possible long-term side effects. The individual risk for the patients is minimal, because of the continuous monitoring of patients and the possibility of unscheduled visits in case of a suspected relapse (suspected by the general practitioner or the investigator). The most common adverse event to be expected is the occurrence of a relapse. In case a relapse occurs (or is suspected) patients undergo an unscheduled clinical visit. In case a relapse is confirmed, patients terminate the active phase of the study and are treated in accordance of current guidelines (e.g. by re-administration of the biologic agent). It can be expected that most patients re-achieve remission thereafter [11,13]. The additional collection of blood for biomarker analysis is not expected to have any influence on blood circulation.

# 10.: Publication

Results are published according to the declaration of Helsinki. Privacy is protected for each patient. The trial will be registered before the first patient is recruited.

# 11.: References

1 Smolen JS, Aletaha D, Koeller M, et al. New therapies for treatment of rheumatoid arthritis. Lancet 2007;370:1861–74. doi:10.1016/S0140-6736(07)60784-3

2 Gossec L, Smolen JS, Gaujoux-Viala C, et al. European League Against Rheumatism recommendations for the management of psoriatic arthritis with pharmacological therapies. Ann Rheum Dis 2012;71:4–12. doi:10.1136/annrheumdis-2011-200350

3 Smolen JS, Landewe R, Breedveld FC, et al. EULAR recommendations for the management of rheumatoid arthritis with synthetic and biological disease-modifying antirheumatic drugs. Ann Rheum Dis 2010;69:964–75. doi:10.1136/ard.2009.126532; 10.1136/ard.2009.126532

4 Smolen JS, Landewé R, Breedveld FC, et al. EULAR recommendations for the management of rheumatoid arthritis with synthetic and biological disease-modifying antirheumatic drugs: 2013 update. Ann Rheum Dis 2014;73:492–509. doi:10.1136/annrheumdis-2013-204573

5 Felson DT, Smolen JS, Wells G, et al. American College of Rheumatology/European League against Rheumatism provisional definition of remission in rheumatoid arthritis for clinical trials. Ann Rheum Dis 2011;70:404–13. doi:10.1136/ard.2011.149765

6 Knevel R, Schoels M, Huizinga TW, et al. Current evidence for a strategic approach to the management of rheumatoid arthritis with disease-modifying antirheumatic drugs: a systematic literature review informing the EULAR recommendations for the management of rheumatoid arthritis. Ann Rheum Dis 2010;69:987–94. doi:10.1136/ard.2009.126748; 10.1136/ard.2009.126748

7 Nam JL, Ramiro S, Gaujoux-Viala C, et al. Efficacy of biological disease-modifying antirheumatic drugs: a systematic literature review informing the 2013 update of the EULAR recommendations for the management of rheumatoid arthritis. Ann Rheum Dis Published Online First: 7 January 2014. doi:10.1136/annrheumdis-2013-204577

8 Buch MH, Emery P. New therapies in the management of rheumatoid arthritis. Curr Opin Rheumatol 2011;23:245–51. doi:10.1097/BOR.0b013e3283454124

9 ten Wolde S, Breedveld FC, Hermans J, et al. Randomised placebo-controlled study of stopping second-line drugs in rheumatoid arthritis. Lancet 1996;347:347–52.

10 ten Wolde S, Hermans J, Breedveld FC, et al. Effect of resumption of second line drugs in patients with rheumatoid arthritis that flared up after treatment discontinuation. Ann Rheum Dis 1997;56:235–9.

11 Tanaka Y, Takeuchi T, Mimori T, et al. Discontinuation of infliximab after attaining low disease activity in patients with rheumatoid arthritis: RRR (remission induction by Remicade in RA) study. Ann Rheum Dis 2010;69:1286–91. doi:10.1136/ard.2009.121491

12 Smolen JS, Nash P, Durez P, et al. Maintenance, reduction, or withdrawal of etanercept after treatment with etanercept and methotrexate in patients with moderate rheumatoid arthritis (PRESERVE): a randomised controlled trial. Lancet 2013;381:918–29. doi:10.1016/S0140-6736(12)61811-X

13 Tanaka Y, Hirata S, Kubo S, et al. Discontinuation of adalimumab after achieving remission in patients with established rheumatoid arthritis: 1-year outcome of the HONOR study. Ann Rheum Dis Published Online First: 28 December 2013. doi:10.1136/annrheumdis-2013-204016

14 Klarenbeek NB, van der Kooij SM, Güler-Yüksel M, et al. Discontinuing treatment in patients with rheumatoid arthritis in sustained clinical remission: exploratory analyses from the BeSt study. Ann Rheum Dis 2011;70:315–9. doi:10.1136/ard.2010.136556

15 Huizinga TWJ, Conaghan PG, Martin-Mola E, et al. Clinical and radiographic outcomes at 2 years and the effect of tocilizumab discontinuation following sustained remission in the second and third year of the ACT-RAY study. Ann Rheum Dis 2015;74:35–43. doi:10.1136/annrheumdis-2014-205752

16 Takeuchi T, Matsubara T, Ohta S, et al. Biologic-free remission of established rheumatoid arthritis after discontinuation of abatacept: a prospective, multicentre, observational study in Japan. Rheumatology (Oxford) 2015;54:683–91. doi:10.1093/rheumatology/keu338

17 Schirmer M, Duftner C, Schmidt WA, et al. Ultrasonography in inflammatory rheumatic disease: an overview. Nat Rev Rheumatol 2011;7:479–88. doi:10.1038/nrrheum.2011.95

18 Aletaha D, Neogi T, Silman AJ, et al. 2010 Rheumatoid arthritis classification criteria: an American College of Rheumatology/European League Against Rheumatism collaborative initiative. Arthritis Rheum 2010;62:2569–81. doi:10.1002/art.27584

19 Colebatch AN, Edwards CJ, Ostergaard M, et al. EULAR recommendations for the use of imaging of the joints in the clinical management of rheumatoid arthritis. Ann Rheum Dis Published Online First: 2013. doi:10.1136/annrheumdis-2012-203158

20 Filer A, de Pablo P, Allen G, et al. Utility of ultrasound joint counts in the prediction of rheumatoid arthritis in patients with very early synovitis. Ann Rheum Dis 2011;70:500–7. doi:10.1136/ard.2010.131573

21 Scire CA, Montecucco C, Codullo V, et al. Ultrasonographic evaluation of joint involvement in early rheumatoid arthritis in clinical remission: power Doppler signal predicts short-term relapse. Rheumatology (Oxford) 2009;48:1092–7. doi:10.1093/rheumatology/kep171

22 Saleem B, Keen H, Goeb V, et al. Patients with RA in remission on TNF blockers: when and in whom can TNF blocker therapy be stopped? Ann Rheum Dis 2010;69:1636–42. doi:10.1136/ard.2009.117341; 10.1136/ard.2009.117341

23 Brown AK, Quinn MA, Karim Z, et al. Presence of significant synovitis in rheumatoid arthritis patients with disease-modifying antirheumatic drug-induced clinical remission: evidence from an imaging study may explain structural progression. Arthritis Rheum 2006;54:3761–73. doi:10.1002/art.22190

24 Peluso G, Michelutti A, Bosello S, et al. Clinical and ultrasonographic remission determines different chances of relapse in early and long standing rheumatoid arthritis. Ann Rheum Dis 2011;70:172–5. doi:10.1136/ard.2010.129924; 10.1136/ard.2010.129924

25 Dejaco C, Duftner C, Wipfler-Freissmuth E, et al. Ultrasound-Defined Remission and Active Disease in Rheumatoid Arthritis: Association with Clinical and Serologic Parameters. Semin Arthritis Rheum. 2012;41:761–7.

26 Mandl P, Balint P V, Brault Y, et al. Clinical and ultrasound-based composite disease activity indices in rheumatoid arthritis: results from a multicenter, randomized study. Arthritis Care Res (Hoboken) 2013;65:879–87. doi:http://dx.doi.org/10.1002/acr.21913

27 Balsa A. Superiority of SDAI over DAS-28 in assessment of remission in rheumatoid arthritis patients using power Doppler ultrasonography as a gold standard. 2010;49:683–90.

28 Sakellariou G, Scirè CA, Verstappen SMM, et al. In patients with early rheumatoid arthritis, the new ACR/EULAR definition of remission identifies patients with persistent absence of functional disability and suppression of ultrasonographic synovitis. Ann Rheum Dis 2013;72:245–9. doi:10.1136/annrheumdis-2012-201817

29 Mandl P, Balint P V, Brault Y, et al. Metrologic properties of ultrasound versus clinical evaluation of synovitis in rheumatoid arthritis: results of a multicenter, randomized study. Arthritis Rheum 2012;64:1272–82. doi:10.1002/art.33491;10.1002/art.33491

30 Marhadour T, Jousse-Joulin S, Chales G, et al. Reproducibility of joint swelling assessments in long-lasting rheumatoid arthritis: influence on Disease Activity Score-28 values (SEA-Repro study part I). J Rheumatol 2010;37:932–7. doi:10.3899/jrheum.090879; 10.3899/jrheum.090879

31 Jousse-Joulin S, d’Agostino MA, Marhadour T, et al. Reproducibility of joint swelling assessment by sonography in patients with long-lasting rheumatoid arthritis (SEA-Repro study part II). J Rheumatol 2010;37:938–45. doi:10.3899/jrheum.090881; 10.3899/jrheum.090881

32 Larché MJ, Seymour M, Lim A, et al. Quantitative power Doppler ultrasonography is a sensitive measure of metacarpophalangeal joint synovial vascularity in rheumatoid arthritis and declines significantly following a 2-week course of oral low-dose corticosteroids. J Rheumatol 2010;37:2493–501. doi:10.3899/jrheum.100322

33 Hammer HB, Sveinsson M, Kongtorp AK, et al. A 78-joints ultrasonographic assessment is associated with clinical assessments and is highly responsive to improvement in a longitudinal study of patients with rheumatoid arthritis starting adalimumab treatment. Ann Rheum Dis 2010;69:1349–51. doi:10.1136/ard.2009.126995; 10.1136/ard.2009.126995

34 Døhn UM, Ejbjerg B, Boonen A, et al. No overall progression and occasional repair of erosions despite persistent inflammation in adalimumab-treated rheumatoid arthritis patients: results from a longitudinal comparative MRI, ultrasonography, CT and radiography study. Ann Rheum Dis 2011;70:252–8. doi:10.1136/ard.2009.123729

35 Haavardsholm EA, Ostergaard M, Hammer HB, et al. Monitoring anti-TNFalpha treatment in rheumatoid arthritis: responsiveness of magnetic resonance imaging and ultrasonography of the dominant wrist joint compared with conventional measures of disease activity and structural damage. Ann Rheum Dis 2009;68:1572–9. doi:10.1136/ard.2008.091801; 10.1136/ard.2008.091801

36 Kawashiri S-Y, Suzuki T, Nakashima Y, et al. Confirmation of effectiveness of tocilizumab by ultrasonography and magnetic resonance imaging in biologic agent-naïve early-stage rheumatoid arthritis patients. Mod Rheumatol 2015;25:948–53. doi:10.3109/14397595.2013.844394

37 D’Agostino M-A, Wakefield RJ, Berner-Hammer H, et al. Value of ultrasonography as a marker of early response to abatacept in patients with rheumatoid arthritis and an inadequate response to methotrexate: results from the APPRAISE study. Ann Rheum Dis Published Online First: 20 November 2015. doi:10.1136/annrheumdis-2015-207709

38 Vastesaeger N, Xu S, Aletaha D, et al. A pilot risk model for the prediction of rapid radiographic progression in rheumatoid arthritis. Rheumatology (Oxford) 2009;48:1114–21. doi:10.1093/rheumatology/kep155; 10.1093/rheumatology/kep155

39 Visser K, Goekoop-Ruiterman YPM, de Vries-Bouwstra JK, et al. A matrix risk model for the prediction of rapid radiographic progression in patients with rheumatoid arthritis receiving different dynamic treatment strategies: post hoc analyses from the BeSt study. Ann Rheum Dis 2010;69:1333–7. doi:10.1136/ard.2009.121160

40 Kamishima T, Sagawa A, Tanimura K, et al. Semi-quantitative analysis of rheumatoid finger joint synovitis using power Doppler ultrasonography: when to perform follow-up study after treatment consisting mainly of antitumor necrosis factor alpha agent. Skeletal Radiol 2010;39:457–65. doi:10.1007/s00256-009-0824-5; 10.1007/s00256-009-0824-5

41 Ellegaard K, Christensen R, Torp-Pedersen S, et al. Ultrasound Doppler measurements predict success of treatment with anti-TNF-&alpha; drug in patients with rheumatoid arthritis: a prospective cohort study. Rheumatology (Oxford) 2011;50:506–12. doi:10.1093/rheumatology/keq336; 10.1093/rheumatology/keq336

42 Bajaj S, Lopez-Ben R, Oster R, et al. Ultrasound detects rapid progression of erosive disease in early rheumatoid arthritis: a prospective longitudinal study. Skeletal Radiol 2007;36:123–8. doi:10.1007/s00256-006-0196-z

43 Fukae J, Kon Y, Henmi M, et al. Change of synovial vascularity in a single finger joint assessed by power doppler sonography correlated with radiographic change in rheumatoid arthritis: comparative study of a novel quantitative score with a semiquantitative score. Arthritis Care Res (Hoboken) 2010;62:657–63. doi:10.1002/acr.20110; 10.1002/acr.20110

44 Bøyesen P, Haavardsholm EA, van der Heijde D, et al. Prediction of MRI erosive progression: a comparison of modern imaging modalities in early rheumatoid arthritis patients. Ann Rheum Dis 2011;70:176–9. doi:10.1136/ard.2009.126953

45 Naredo E, Collado P, Cruz A, et al. Longitudinal power Doppler ultrasonographic assessment of joint inflammatory activity in early rheumatoid arthritis: predictive value in disease activity and radiologic progression. Arthritis Rheum 2007;57:116–24. doi:10.1002/art.22461

46 Foltz V, Gandjbakhch F, Etchepare F, et al. Power Doppler ultrasound, but not low-field magnetic resonance imaging, predicts relapse and radiographic disease progression in rheumatoid arthritis patients with low levels of disease activity. Arthritis Rheum 2012;64:67–76. doi:10.1002/art.33312; 10.1002/art.33312

47 Saleem B, Brown AK, Quinn M, et al. Can flare be predicted in DMARD treated RA patients in remission, and is it important? A cohort study. Ann Rheum Dis 2012;71:1316–21. doi:10.1136/annrheumdis-2011-200548

48 Gärtner M, Mandl P, Radner H, et al. Sonographic joint assessment in rheumatoid arthritis: associations with clinical joint assessment during a state of remission. Arthritis Rheum 2013;65:2005–14. doi:10.1002/art.38016

49 Anandarajah A, Thiele R, Giampoli E, et al. Patients with rheumatoid arthritis in clinical remission manifest persistent joint inflammation on histology and imaging studies. J Rheumatol 2014;41:2153–60. doi:10.3899/jrheum.140411

50 Iwamoto T, Ikeda K, Hosokawa J, et al. Ultrasonographic assessment predicts relapse after discontinuation of biological agents in patients with rheumatoid arthritis in clinical remission. Arthritis Care Res (Hoboken) Published Online First: 10 February 2014. doi:10.1002/acr.22303

51 Naredo E, Valor L, De la Torre I, et al. Predictive value of Doppler ultrasound-detected synovitis in relation to failed tapering of biologic therapy in patients with rheumatoid arthritis. Rheumatology (Oxford) Published Online First: 1 March 2015. doi:10.1093/rheumatology/kev006

52 Aletaha D, Alasti F, Smolen JS. Rheumatoid factor determines structural progression of rheumatoid arthritis dependent and independent of disease activity. Ann Rheum Dis 2013;72:875–80. doi:10.1136/annrheumdis-2012-201517

53 Machold KP, Stamm TA, Nell VPK, et al. Very recent onset rheumatoid arthritis: clinical and serological patient characteristics associated with radiographic progression over the first years of disease. Rheumatology (Oxford) 2007;46:342–9. doi:10.1093/rheumatology/kel237

54 Turesson C, Jacobsson LTH, Sturfelt G, et al. Rheumatoid factor and antibodies to cyclic citrullinated peptides are associated with severe extra-articular manifestations in rheumatoid arthritis. Ann Rheum Dis 2007;66:59–64. doi:10.1136/ard.2006.054445

55 Böhler C, Radner H, Smolen JS, et al. Serological changes in the course of traditional and biological disease modifying therapy of rheumatoid arthritis. Ann Rheum Dis 2013;72:241–4. doi:10.1136/annrheumdis-2012-202297

56 Renaudineau Y, Jamin C, Saraux A, et al. Rheumatoid factor on a daily basis. Autoimmunity 2005;38:11–6. doi:10.1080/08916930400022574

57 Winchester RJ, Agnello V, Kunkel HG. Gamma globulin complexes in synovial fluids of patients with rheumatoid arthritis. Partial characterization and relationship to lowered complement levels. Clin Exp Immunol 1970;6:689–706.http://www.pubmedcentral.nih.gov/articlerender.fcgi?artid=1712723&tool=pmcentrez&rendertype=abstract (accessed 3 Feb2016).

58 Taylor RP, Lindorfer MA. Drug insight: the mechanism of action of rituximab in autoimmune disease--the immune complex decoy hypothesis. Nat Clin Pract Rheumatol 2007;3:86–95. doi:10.1038/ncprheum0424

59 Aletaha D, Alasti F, Smolen JS. Rheumatoid factor, not antibodies against citrullinated proteins, is associated with baseline disease activity in rheumatoid arthritis clinical trials. Arthritis Res Ther 2015;17:229. doi:10.1186/s13075-015-0736-9

60 Sabharwal UK, Vaughan JH, Fong S, et al. Activation of the classical pathway of complement by rheumatoid factors. Assessment by radioimmunoassay for C4. Arthritis Rheum 1982;25:161–7.http://www.ncbi.nlm.nih.gov/pubmed/7066047 (accessed 3 Feb2016).

61 Kiener HP, Baghestanian M, Dominkus M, et al. Expression of the C5a receptor (CD88) on synovial mast cells in patients with rheumatoid arthritis. Arthritis Rheum 1998;41:233–45. doi:10.1002/1529-0131(199802)41:2<233::AID-ART7>3.0.CO;2-V

62 Ravetch J V, Bolland S. IgG Fc receptors. Annu Rev Immunol 2001;19:275–90. doi:10.1146/annurev.immunol.19.1.275

63 Dörner T, Radbruch A, Burmester GR. B-cell-directed therapies for autoimmune disease. Nat Rev Rheumatol 2009;5:433–41. doi:10.1038/nrrheum.2009.141

64 Blüml S, McKeever K, Ettinger R, et al. B-cell targeted therapeutics in clinical development. Arthritis Res Ther 2013;15 Suppl 1:S4. doi:10.1186/ar3906

65 Montecino-Rodriguez E, Dorshkind K. B-1 B cell development in the fetus and adult. Immunity 2012;36:13–21. doi:10.1016/j.immuni.2011.11.017

66 van der Heijde DM. Plain X-rays in rheumatoid arthritis: overview of scoring methods, their reliability and applicability. Baillière’s Clin Rheumatol 1996;10:435–53.http://www.ncbi.nlm.nih.gov/pubmed/8876953 (accessed 4 Feb2016).

67 Bredemeier M, Campos GG, de Oliveira FK. Updated systematic review and meta-analysis of randomized controlled trials comparing low- versus high-dose rituximab for rheumatoid arthritis. Clin Rheumatol 2015;34:1801–5. doi:10.1007/s10067-015-2977-z

68 Valleala H, Korpela M, Hienonen-Kempas T, et al. Long-term real-life experience with rituximab in adult Finnish patients with rheumatoid arthritis refractory or with contraindication to anti-tumor necrosis factor drugs. J Clin Rheumatol 2015;21:24–30. doi:10.1097/RHU.0000000000000166

69 Backhaus M, Ohrndorf S, Kellner H, et al. Evaluation of a novel 7-joint ultrasound score in daily rheumatologic practice: a pilot project. Arthritis Rheum 2009;61:1194–201. doi:10.1002/art.24646

70 Backhaus TM, Ohrndorf S, Kellner H, et al. The US7 score is sensitive to change in a large cohort of patients with rheumatoid arthritis over 12 months of therapy. Ann Rheum Dis 2013;72:1163–9. doi:10.1136/annrheumdis-2012-201397

71 Scheel AK, Hermann KG, Kahler E, et al. A novel ultrasonographic synovitis scoring system suitable for analyzing finger joint inflammation in rheumatoid arthritis. Arthritis Rheum 2005;52:733–43. doi:10.1002/art.20939

72 Nell VPK, Machold KP, Stamm TA, et al. Autoantibody profiling as early diagnostic and prognostic tool for rheumatoid arthritis. Ann Rheum Dis 2005;64:1731–6. doi:10.1136/ard.2005.035691

73 Jansen ALMA, van der Horst-Bruinsma I, van Schaardenburg D, et al. Rheumatoid factor and antibodies to cyclic citrullinated Peptide differentiate rheumatoid arthritis from undifferentiated polyarthritis in patients with early arthritis. J Rheumatol 2002;29:2074–6.http://www.ncbi.nlm.nih.gov/pubmed/12375314 (accessed 4 Feb2016).

74 Backhaus M, Burmester GR, Gerber T, et al. Guidelines for musculoskeletal ultrasound in rheumatology. Ann Rheum Dis 2001;60:641–9.

75 Prevoo ML, van ’t Hof MA, Kuper HH, et al. Modified disease activity scores that include twenty-eight-joint counts. Development and validation in a prospective longitudinal study of patients with rheumatoid arthritis. Arthritis Rheum 1995;38:44–8.http://www.ncbi.nlm.nih.gov/pubmed/7818570 (accessed 10 Feb2016).

76 Smolen JS, van der Heijde D, Machold KP, et al. Proposal for a new nomenclature of disease-modifying antirheumatic drugs. Ann Rheum Dis 2014;73:3–5. doi:10.1136/annrheumdis-2013-204317
