# Supplementary material for: Ultrasound-based withdrawal of biologics in rheumatoid arthritis (RA-BioStop)
Source: RMD Open. 2026 Aug 7;12(3):e007003. doi: 10.1136/rmdopen-2026-007003 (PMC13475499; doi:10.1136/rmdopen-2026-007003)
Supplement: Supplementary file 2 [file rmdopen-12-3-s002.docx]

**Supplementary File 2**

**Supplementary Table 1**: Allowed and prohibited medication:

| **Allowed medication** | **Prohibited medication** |
| --- | --- |
| Conventional synthetic DMARD at a stable dose | Oral, intramuscular or intravenous corticosteroids |
| Analgesics (incl. NSAID at a stable dose) | Intraarticular injections (corticosteroids, local anaesthetics) in joints assessed by sonography or within the 28-joint count |
| Drugs to treat concomitant medical conditions (e.g. hypertension) | New immunosuppressive drugs |
| Intraarticular injections (corticosteroids, local anaesthetics) in joints not investigated by sonography or within the 28-joint count | Chemotherapeutic agents |
|  | Rituximab, other biological agents or targeted synthetic DMARDs for any reason |

Overview of allowed and prohibited medication in the active phase of the study.

DMARD, Disease-Modifying Antirheumatic Drug; NSAID, Non-Steroidal Anti-Inflammatory Drug;

**Supplementary Table 2:** Ultrasound protocol and machine settings:

| Assessed joints bilaterally | - Wrist - 2nd and 3rd metacarpophalangeal - 2nd and 3rd proximal interphalangeal - 2nd and 5th metatarsophalangeal |
| --- | --- |
| Scanning plans and joint aspects | Transverse and longitudinal scans of the dorsal and palmar aspect of each joint |
| Ultrasound machine | MyLab Twice (Esaote, Genova, Italy) |
| Ultrasound transducers | LA435 (6–18 MHz), LA523 (4-13 MHz) |
| B-mode image optimization | - Frequencies of 18.0 MHz as well as 10-13 MHz were used for B-mode ultrasound, respectively - Imaging parameters were adjusted to maximize contrast between examined structures |
| Doppler-settings | - Frequency of 9.1 MHz (small joints, entheses) or 6.3 (large joints) - Pulse repetition frequency: 750 Hz; medium persistence. - Doppler-gain was optimized by increasing gain until noise appeared and then reduced just enough to suppress the noise |

**Supplementary Table 3.** Patients’ characteristics grouped according to the presence of Power Doppler signs of synovitis at baseline

|  | **PD+** | | **PD-** |  | **p-value*** |
| --- | --- | --- | --- | --- | --- |
| Variable |  | N |  | N |  |
| Female, n (%) | 23 (76.7) | 30 | 5 (62.5) | 8 | 0.411 |
| Age [years], mean (SD) | 60.4 (10.8) | 30 | 53.6 (15.8) | 8 | 0.392 |
| Current smoker, n (%)  Previous smoker, n (%)  Packyears, mean (SD) | 11 (36.7)  7 (23.3)  20.6 (18.6) | 30  30  18 | 1 (12.5)  5 (62.5)  1.8 (2.2) | 8  8  4 | 0.159  **0.007** |
| RF+ at diagnosis, n (%) | 11 (52.4) | 21 | 1 (50.0) | 2 | >0.999 |
| RF+ at baseline, n (%) | 10 (62.5) | 16 | 1 (100) | 1 | >0.999 |
| CCP+ at diagnosis, n (%) | 17 (89.5) | 19 | 3 (100) | 3 | >0.999 |
| CCP+ at baseline, n (%) | 18 (94.7) | 19 | 2 (100) | 2 | >0.999 |
| Disease duration [years], mean (SD) | 14.0 (10.0) | 30 | 8.2 (4.4) | 8 | 0.097 |
| Duration of remission [months], mean (SD) | 21.0 (13.5) | 30 | 17.8 (11.7) | 8 | 0.519 |
| Tender joint count (28-joints), median (range) | 0 (0-2) | 30 | 0 (0-1) | 8 | 0.720 |
| Swollen joint count (28-joints), median (range) | 0 (0-1) | 30 | 0 (0-1) | 8 | >.999 |
| Patient pain assessment (VAS) [mm], mean (SD) | 5.5 (10.3) | 30 | 8.6 (9.6) | 8 | 0.242 |
| Patient global assessment (VAS) [mm], mean (SD) | 6.0 (9.3) | 30 | 9.4 (10.1) | 8 | 0.292 |
| Evaluator global assessment (VAS) [mm], mean (SD) | 3.2 (5.6) | 30 | 2.8 (4.5) | 8 | 0.884 |
| CRP [mg/L], mean (SD) | 2.9 (4.1) | 30 | 1.1 (1.3) | 8 | 0.046 |
| ESR [mm/h], mean (SD) | 18.2 (14.3) | 28 | 9.7 (9.4) | 7 | 0.136 |
| DAS-28, mean (SD) | 2.0 (0.9) | 28 | 1.5 (0.8) | 7 | 0.217 |
| CDAI, mean (SD) | 1.3 (1.6) | 30 | 1.5 (2.0) | 8 | 0.936 |
| SDAI, mean (SD) | 1.6 (1.7) | 30 | 1.6 (1.9) | 8 | 0.759 |
| HAQ, mean (SD) | 0.2 (0.3) | 22 | 0.1 (0.2) | 6 | 0.297 |
| Biologic DMARDs at time of inclusion into the study  Infliximab, n (%)  Etanercept, n (%)  Adalimumab, n (%)  Golimumab, n (%)  Certolizumab pegol, n (%)  Abatacept, n (%)  Tocilizumab, n (%) | 4 (13.3%)  8 (26.7%)  7 (23.3%)  5 (16.7%)  4 (13.3%)  1 (3.3%)  1 (3.3%) | 30 | 0  3 (37.5%)  2 (25.0%)  2 (25.0%)  0  1 (12.5%)  0 | 8 | n.d. |
| Extended^¥^ administration interval of biologic agent at inclusion into the study | 14 (46.7) | 30 | 2 (25.0) | 8 | 0.426 |
| Conventional synthetic DMARDs at baseline*  Leflunomide, n  Methotrexate, n  Methotrexate + Sulfasalazine, n  no conventional synthetic DMARD | 6 (20.0)  21 (70.0)  1 (3.3)  2 (6.7) | 30 | 2 (25.0)  6 (75.0)  0  0 | 8 | n.d. |

*crude p-values are depicted, none of the differences were statistically significant after Bonferroni correction for multiple testing

^¥^as compared to the administration interval recommended in the Summary of Product Characteristics

CCP, anti-cyclic citrullinated peptide antibodies; CDAI, clinical disease activity index;

CRP, C-reactive protein; DAS-28, disease activity score 28; DMARD, disease modifying anti-rheumatic drug; ESR, erythrocyte sedimentation rate; HAQ, health assessment questionnaire; n, number; n.d., p-value not determined; RF, rheumatoid factor; SD, standard deviation; SDAI, simplified disease activity index;

**Supplementary table 4:** Administration intervals of biological disease modifying anti-rheumatic drugs (bDMARDs)

|  | n | Mean interval (days) | Standard deviation (days) |
| --- | --- | --- | --- |
| Infliximab (intravenous) | 4 | 52 | 20.9 |
| Etanercept (subcutaneous) | 11 | 8.5 | 5.7 |
| Adalimumab (subcutaneous) | 9 | 24.9 | 9.3 |
| Golimumab (subcutaneous) | 7 | 30.6 | 5.1 |
| Certolizumab pegol (subcutaneous) | 4 | 19.3 | 10.5 |
| Abatacept (subcutaneous) | 1 | 7.0 | 0.0 |
| Abatacept (intravenous) | 1 | 35.0 | 0.0 |
| Tocilicumab (intravenous) | 1 | 32.0 | 0.0 |

Administration intervals of bDMARDs indicated as mean in days with standard deviation

**Supplementary table 5:** Adverse events

| **Event** | **N** |
| --- | --- |
| Respiratory tract infection | 3 |
| Chronic obstructive pulmonary disease (COPD) flare | 1 |
| Mechanic shoulder problem | 2 |
| Episode of allergic rhinitis | 1 |
| New thyroid nodule | 1 |
| Mild elevation of liver enzymes | 1 |

**Supplementary table 6**: Explorative secondary outcomes

|  | Relapse until week 16 | Relapse until week 24 | Relapse until week 52 |
| --- | --- | --- | --- |
| Median (range) BL PD score in patients with relapse | 3.0 (1.0 – 15.0) | 2.5 (0.0 – 15.0) | 2.0 (0.0 – 15.0) |
| Median (range) BL PD score in patients without relapse | 1.0 (0.0 – 14.0) | 1.0 (0.0 – 14.0) | 1.0 (0.0 – 14.0) |
| OR (95%CI) for each additional point of the PD score at BL | 1.2 (0.98-1.43) | 1.2 (0.96-1.37) | 1.1 (0.93-1.32) |
| Mean (SD) BL GSS in patient with relapse | 9.0 (6.2) | 11.6 (10.1) | 10.6 (9.6) |
| Mean (SD) BL GSS in patient without relapse | 11.1 (7.4) | 10.3 (5.8) | 10.6 (5.6) |
| Median (range) residual SJ at BL in patients with relapse | 0.0 (0.0-0.0) | 0.0 (0.0-1.0) | 0.0 (0.0-1.0) |
| Median (range) residual SJ at BL in patients without relapse | 0.0 (0.0-1.0) | 0.0 (0.0-1.0) | 0.0 (0.0-1.0) |
| Median (range) residual TJ at BL in patients with relapse | 0.0 (0.0-2.0) | 0.0 (0.0-2.0) | 0.0 (0.0-2.0) |
| Median (range) residual TJ at BL in patients without relapse | 0.0 (0.0-1.0) | 0.0 (0.0-1.0) | 0.0 (0.0-1.0) |
| RR (95% CI) for future relapse in patients with residual SJ at BL | 0.4 (0.0-5.2) | 0.9 (0.2-5.5) | 0.7 (0.1-4.0) |
| RR (95% CI) for future relapse in patients with residual TJ at BL | 1.2 (0.3-4.7) | 1.8 (0.6-5.4) | 2.0 (0.8-4.4) |

Exploratory secondary outcomes at baseline in patients with and without relapse until 16, 24, 52 weeks, respectively.

BL, baseline; CI, confidence interval; GSS, Grey scale synovitis; OR, odds ratio; PD, Power Doppler; RR, relative risk; SD, standard deviation, SJ, swollen joint; TJ, tender joint;

**Supplementary Figure 1.** Kaplan-Meier analysis of relapses according to presence or absence of at least one swollen (A) or tender (B) joint out of a 28-joint count at baseline.

A. Swollen joint count >0 at baseline

B. Tender joint count >0 at baseline
